# Comparison of in vitro growth characteristics of *Cryptosporidium hominis* (IdA15G1) and *Cryptosporidium parvum* (Iowa-IIaA17G2R1 and IIaA18G3R1)

**DOI:** 10.1007/s00436-023-07979-0

**Published:** 2023-09-30

**Authors:** Samantha Gunasekera, Peta L. Clode, Brendon King, Paul Monis, Benjamin Thierry, Jillian M. Carr, Abha Chopra, Mark Watson, Mark O’Dea, Nawal Hijjawi, Una Ryan

**Affiliations:** 1 Harry Butler Institute, College of Environmental and Life Sciences, Murdoch University, Murdoch, Western Australia 6150 Australia; 2 Centre for Microscopy, Characterisation, and Analysis and School of Biological Sciences, The University of Western Australia, Crawley, Western Australia 6009 Australia; 3 South Australian Water Corporation, Adelaide, South Australia 5000 Australia; 4 Future Industries Institute, University of South Australia, Adelaide, South Australia 5095 Australia; 5 College of Medicine and Public Health, Flinders Health and Medical Research Institute, Flinders University, Bedford Park, South Australia 5042 Australia; 6 Immunology and Infectious Diseases, Murdoch University, Murdoch, Western Australia 6150 Australia; 7 Department of Medical Laboratory Sciences, Faculty of Applied Health Sciences, The Hashemite University, P.O. Box 150459, Zarqa, 13115 Jordan

**Keywords:** *Cryptosporidium*, Life cycle, In vitro, Scanning electron microscopy, *C. hominis*, *C. parvum*

## Abstract

*Cryptosporidium* is a major cause of diarrhoeal disease and mortality in young children in resource-poor countries, for which no vaccines or adequate therapeutic options are available. Infection in humans is primarily caused by two species: *C. hominis* and *C. parvum*. Despite *C. hominis* being the dominant species infecting humans in most countries, very little is known about its growth characteristics and life cycle in vitro, given that the majority of our knowledge of the in vitro development of *Cryptosporidium* has been based on *C. parvum*. In the present study, the growth and development of two *C. parvum* isolates (subtypes Iowa-IIaA17G2R1 and IIaA18G3R1) and one *C. hominis* isolate (subtype IdA15G1) in HCT-8 cells were examined and compared at 24 h and 48 h using morphological data acquired with scanning electron microscopy. Our data indicated no significant differences in the proportion of meronts or merozoites between species or subtypes at either time-point. Sexual development was observed at the 48-h time-point across both species through observations of both microgamonts and macrogamonts, with a higher frequency of macrogamont observations in *C. hominis* (IdA15G1) cultures at 48-h post-infection compared to both *C. parvum* subtypes. This corresponded to differences in the proportion of trophozoites observed at the same time point. No differences in proportion of microgamonts were observed between the three subtypes, which were rarely observed across all cultures. In summary, our data indicate that asexual development of *C. hominis* is similar to that of *C. parvum,* while sexual development is accelerated in *C. hominis.* This study provides new insights into differences in the in vitro growth characteristics of *C. hominis* when compared to *C. parvum*, which will facilitate our understanding of the sexual development of both species.

## Introduction

Enteric protozoan parasites of the *Cryptosporidium* genus are a major cause of diarrhoea-related morbidity and mortality, especially in young children, with higher endemicity occurring in resource-poor countries (Yang et al. 2021). In industrialised countries, the burden of cryptosporidiosis is primarily associated with waterborne outbreaks (Gharpure et al. 2019; Ryan et al. 2022). Currently, at least 45 *Cryptosporidium* species are recognised (Prediger et al. 2021; Ryan et al. 2021), with two main species, *C. hominis* and *C. parvum*, responsible for over 90% of infections reported in humans globally (Chalmers et al. 2019; Feng et al. 2018; Ryan et al. 2021). While most species of *Cryptosporidium* exhibit a high level of host specificity, *C. parvum* is one of the few species that has a broad host range (Feng et al. 2018). Zoonotic transmission of *C. parvum* is common and represents an important aspect of the epidemiology of *Cryptosporidium* infections. This contrasts with *C. hominis* which, despite being reported in numerous hosts, is primarily a human pathogen (Widmer et al. 2020; Yang et al. 2021). There is no vaccine to prevent *Cryptosporidium* infection, and effective treatments for cryptosporidiosis are lacking, with only one anti-cryptosporidial drug (nitazoxanide) that is used clinically (Rahman et al. 2022). The efficacy of nitazoxanide for the treatment of cryptosporidiosis in immunocompromised and young patients, who are most vulnerable to severe disease and death from infection, is significantly lower than in immunocompetent patients. This poses a major challenge for mitigating the health burden of human cryptosporidiosis (Diptyanusa and Sari 2021; Khan and Witola 2023).

*Cryptosporidium* has a complex monoxenous life cycle where the infectious stage of the parasite, the oocyst, is ingested by the susceptible host. Each sporulated oocyst contains four sporozoites that are released after the oocyst exits the stomach and reaches the intestinal lumen, where they can subsequently invade the intestinal epithelium from the apical surface (Wetzel et al. 2005). The sporozoites then inhabit a niche within an intracellular but extracytoplasmic location of the host cell and develop a feeder organelle which anchors the parasite to the host epithelial cell (Forney et al. 1999). Upon invasion of the host cell, the sporozoite will rapidly mature into a trophozoite and later undergo merogony to form a meront (Edwinson et al. 2016). The meronts then release uninucleated merozoites into the intestinal lumen where they subsequently invade surrounding intestinal epithelial cells (Guérin et al. 2021). Merozoites will continue to cycle through the asexual phases of development (trophozoites, meronts, merozoites) three times, before initiating sexual development through the formation of microgamonts and macrogamonts (English et al. 2022). Fertilisation will then occur, resulting in the formation of new oocysts that are shed in the faeces. Consequently, transmission of *Cryptosporidium* occurs most commonly through the faecal–oral route (Feng et al. 2018).

Since the first report of *Cryptosporidium* cultivation in cell culture (Current and Haynes 1984), great strides have been made in the development and application of tools to both genetically manipulate *Cryptosporidium* and study its life cycle in vitro (Aldeyarbi and Karanis 2016; Borowski et al. 2010; Cardenas et al. 2020; Edwinson et al. 2016; English et al. 2022; Hashim et al. 2004; Hashim et al. 2006; Heo et al. 2018; Hijjawi et al. 2010; Hijjawi et al. 2001; Huang et al. 2004; Jumani et al. 2019; Mauzy et al. 2012; Miller et al. 2018; Morada et al. 2016; Pawlowic et al. 2019; Petry et al. 2009; Tandel et al. 2019; Tandel et al. 2023; Varughese et al. 2014; Vinayak et al. 2015; Warren et al. 2008; Wilke et al. 2019). The vast majority of these studies have focused on *C. parvum* due to its wide host range and readily available commercial stocks from animal sources (e.g. from Waterborne Inc, BioPoint Pty Ltd). Comparatively, very few studies on the life cycle and growth characteristics of *C. hominis* have been reported*,* despite being the dominant species infecting humans in most countries (Yang et al. 2021). The aim of this study was to acquire comprehensive morphological data using a scanning electron microscopy (SEM) approach to compare the growth characteristics of *C. hominis* (IdA15G1) and *C. parvum* (Iowa-IIaA17G2R1 and IIaA18G3R1) in a HCT-8 cell-line based in vitro culture system.

## Methods

### Collection, purification, and genotyping of *Cryptosporidium* oocysts

The *C. parvum* (Iowa-IIaA17G2R1) isolate was obtained commercially from BioPoint Pty Ltd (Sydney, Australia). Both the *C. parvum* (IIaA18G3R1) and *C. hominis* (IdA15G1) isolates were obtained from faecal samples from a local public health diagnostic laboratory (Perth, Australia) where *Cryptosporidium* infection was confirmed via microscopy with Ziehl-Neelsen staining. The *C. parvum* samples utilised in this study will be referred to as *C. parvum* (Iowa) and *C. parvum* (clinical isolate) from this point onwards. Oocysts were purified from faecal matter using a procedure outlined previously (Morgan et al. 1995) with the following modifications: omission of the potassium dichromate incubation step, replacement of ether with ethyl acetate (Chem-Supply), and addition of three phosphate-buffered saline (PBS) washes after PBS-ethyl acetate sedimentation. Samples were further purified using Ficoll-density centrifugation (Lumb et al. 1988) with the following modifications: the 4% and 6% Ficoll 400 (Sigma) in PBS (Gibco) containing 16% sodium diatrizoate (MP Bio) were excluded from the discontinuous step gradient, and the centrifugation step was performed for 20 min at 2000 × *g* at 4 °C. Following purification, the patient-derived *Cryptosporidium* oocyst isolates were stored at 4 °C in PBS supplemented with 1% v/v penicillin-streptomycin and 1% v/v amphotericin B (both from Sigma) prior to experiments. The commercially obtained *C. parvum* (Iowa) isolate was stored at 4 °C in PBS with no further purification required. All oocyst isolates were used for experiments within 12 weeks of purification.

All oocyst isolates were genotyped using a nested PCR targeted to the 60-kDa glycoprotein (*gp60*) locus, with primer sets described previously (Peng et al. 2001) and shown in Table 1. DNA was amplified in a 20 μL reaction containing 1× GoTaq Master Mix (Promega), 2.5 mM MgCl_2_, 0.5 mM dNTPs, 0.5 μM forward primer, 0.5 μM reverse primer, and 1 unit GoTaq DNA polymerase (Promega) per reaction. DNA amplification was carried out using a BioRad C1000 Thermal Cycler under the following cycling conditions described previously (Strong et al. 2000): briefly, an initial denaturation cycle was performed at 95 °C for 3 min, followed by 35 cycles of 94 °C for 45 s, 50 °C for 45 s, and 72 °C for 60 s, with a final extension cycle performed at 72 °C for 10 min. Amplicons from the secondary PCR were then run on a 1% agarose gel and subject to Sanger sequencing. The *gp60* subtype was determined using NCBI BLAST and Clustal W alignments against an in-house database.

**Table 1:** *gp60* primer names and sequences used in the present study (Peng et al. 2001)

Primer name	Primer sequence
Primary forward AL 3531	5′-ATAGTCTCCGCTGTATTC-3′
Primary reverse AL 3534	5′-GCAGAGGAACCAGCATC-3′
Secondary forward AL 3532	5′-TCCGCTGTATTCTCAGCC-3′
Secondary reverse AL 3533	5′-GAGATATATCTTGGTGCG-3′

### HCT-8 cell culture

A human epithelial cell line (HCT-8, human ileocecal colorectal adenocarcinoma, ATCC CCL-244) was routinely passaged in 75-cm^2^ culture flasks in RPMI-1640 (Sigma) supplemented with 10% v/v foetal bovine serum (Bovogen), 1% v/v penicillin-streptomycin (Sigma), 2 mM L-glutamine (Sigma), and 15 mM HEPES (Sigma) with pH adjusted to 7.2. The cell line tested negative for mycoplasma prior to experiments, and all experiments were performed between 60 and 100 cell doublings. For infection experiments, cell suspensions were prepared using 0.25% v/v trypsin-EDTA (Sigma), seeded onto 10-mm circular borosilicate glass coverslips at a density of 5.0 ×10^4^ cells per well in 24-well transwell plates, and grown to 70–90% confluence.

### Pre-treatment of oocysts and infection of HCT-8 cells

*Cryptosporidium* oocyst suspensions were quantified via haemocytometer count, pelleted at 1800 × *g* for 10 min, then resuspended in 0.25% v/v sodium hypochlorite and incubated at 4 °C for 30 min. Excystation pre-treatment of the oocysts was performed as described previously (King et al. 2015), omitting the infection medium washing steps after centrifugation. Oocysts were resuspended at a concentration of 1.0 × 10^5^ oocysts mL^−1^ in an infection medium composed of RPMI-1640 (Sigma) supplemented with 2 mM L-glutamine, 5.6 mM glucose, 0.02% w/v bovine bile, 15 mM HEPES, 0.6 μM folic acid, 7.3 μM 4-aminobenzoic acid, 2.1 μM calcium pantothenate, 50 μM L-ascorbic acid, 2.5 μg mL^−1^ amphotericin B, 1% v/v penicillin-streptomycin (all from Sigma), and 1% v/v foetal bovine serum (Bovogen), with pH adjusted to 7.2. The oocyst suspension was immediately applied to HCT-8 cell monolayers at a volume of 500 μL, which corresponded to 5.0 × 10^4^ oocysts per well. Four wells containing HCT-8 monolayers were infected per *Cryptosporidium* subtype for each time-point, and separate plates were used for each time-point to facilitate downstream sample processing. Immediately following oocyst application to monolayers, the plates were centrifuged at 410 × *g* for 5 min at room temperature. Each plate was then incubated at 37 °C and 5% CO_2_ in a humidified cell culture incubator for 5 h, before cell monolayers were gently rinsed with pre-warmed PBS and the infection medium was replaced. The plates were incubated for a further 19 h (for 24-h infections) and 43 h (for 48-h infections), with infection medium refreshed at 24-h post-infection for plates designated for 48-h infections. These two time-points were selected specifically to assess the transition from asexual to sexual development which has been documented to occur in this time-frame (English et al. 2022; Hijjawi et al. 2001; Tandel et al. 2019).

### Sample preparation for scanning electron microscopy

At 24-h and 48-h post-infection, the infection medium was aspirated from each well, and monolayers were rinsed three times with PBS (Gibco). Samples were then chemically fixed in 2.5% v/v Grade I glutaraldehyde (Sigma) in PBS pH 7.4 through overnight incubation at 4 °C. The next morning, the glutaraldehyde was aspirated from each well, and monolayers were rinsed three times with PBS. Samples were dehydrated with analytical reagent grade ethanol (Chem-Supply) at concentrations of 30% v/v, 50% v/v, 70% v/v, and 90% v/v, all diluted with deionised water, with two 5-min incubation periods for each dilution. Three subsequent 7.5-min incubation periods with 100% v/v ethanol immediately followed. Samples underwent further chemical dehydration with hexamethyldisilazane (Sigma) diluted with ethanol at concentrations of 30% v/v, 50% v/v, and 70% v/v, with one 7.5-min incubation period per dilution. Three subsequent 7.5-min incubation periods with 100% v/v hexamethyldisilazane immediately followed, with residual hexamethyldisilazane from the final incubation period allowed to evaporate overnight in the fume hood. Borosilicate glass coverslips containing the sample were then mounted onto 12.5-mm diameter aluminium specimen stubs (Emgrid Australia) using carbon tape, and the edges were sealed with carbon paint. Samples were then sputter coated with 3 nm platinum.

### Scanning electron microscopy image acquisition and data analysis

High resolution images were acquired using a Zeiss 55VP field emission SEM using the in-lens secondary electron detector and an accelerating voltage set to 5 kV. Large-area automated image acquisition was also undertaken using FEI Maps 2.0 software (Thermo Fisher Scientific) on a FEI Verios 460 XHR field emission SEM using the Everhart-Thornley detector with the accelerating voltage set to 5 kV. The FEI Maps 2.0 software was used to set up unsupervised batch data collection from all samples at a magnification of 5000 ×. Data from both electron microscopes was collected from a minimum of three separate infected wells per time-point per subtype. In the case of automated image collection, data was acquired from each sample from a minimum of eight 10 × 10 grids of images (i.e. 100 images), with each image accounting for a 1050 μm^2^ area of the sample. The data acquired from large areas was manually translated into numerical counts of *Cryptosporidium* life cycle stages. Each life cycle stage was identified based on criteria previously described in detail (Borowski et al. 2010). Ambiguous life cycle stages were further evaluated using additional sources (Koh et al. 2014; Pinto et al. 2022). Boxplots of numerical data were generated using ggplot2 v3.4.0 (Wickham 2016).

### Statistical analysis

Statistical analyses were performed using R (v4.1.1) (R Core Team 2021). Data was assessed for normality using a Shapiro-Wilk test and homogeneity of variance using a Levene’s test. Given that these assumptions for parametric testing were not met, a Kruskal-Wallis test with a Dunn’s multiple comparisons test was performed for each life cycle stage at each time-point to assess whether there were any statistically significant differences in these variables between the three subtypes of *Cryptosporidium* that were utilised in this study.

## Results

This study utilised morphological data to compare the in vitro life cycle progression of *C. parvum* (Iowa-IIaA17G2R1 and IIaA18G3R1) and *C. hominis* (IdA15G1) at 24-h and 48-h post-infection. Overall, in vitro life cycle stages were primarily asexual at the 24-h time-point across all three subtypes, and both isolates of *C. parvum* (Iowa and clinical isolate) still predominantly exhibited asexual developmental stages at the 48-h time-point. Notably, sexual life cycle stages were frequently observed at 48-h post-infection in *C. hominis*. At 24-h post-infection, a total of 915 individuals (at different life cycle stages) were counted for *C. hominis*, 828 for *C. parvum* (clinical isolate), and 836 for *C. parvum* (Iowa). At 48-h post-infection, a total of 1281 individuals (at different life cycle stages) were counted for *C. hominis*, 2356 for *C. parvum* (clinical isolate), and 3710 were counted for *C. parvum* (Iowa).

**Fig. 1:**
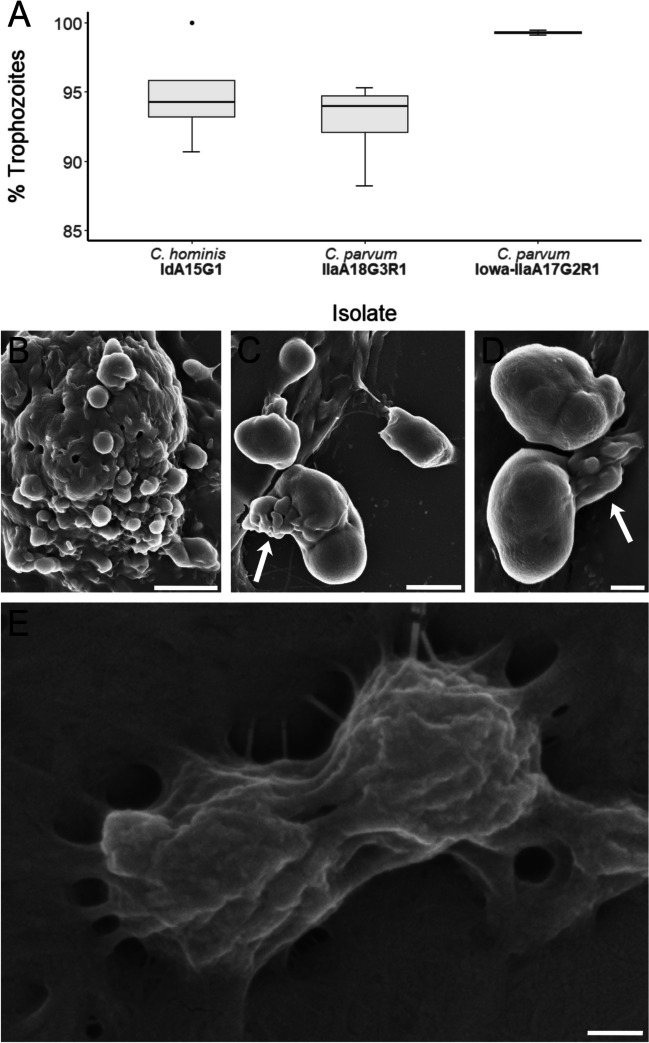
**A** Proportion of trophozoites observed at 24-h post-infection across all three isolates. **B** Scanning electron micrograph of immature *C. hominis* trophozoites at 24-h post-infection (scale bar: 2 μm). **C**–**D** Scanning electron micrographs of mature *C. hominis* trophozoites at 24-h post-infection with feeder organelles indicated by arrows (scale bars: C = 2 μm, D = 1 μm). **E** Scanning electron micrograph of *C. hominis* trophozoites in lateral pairing observed at 48-h post-infection (scale bar: 200 nm)

**Fig. 2:**
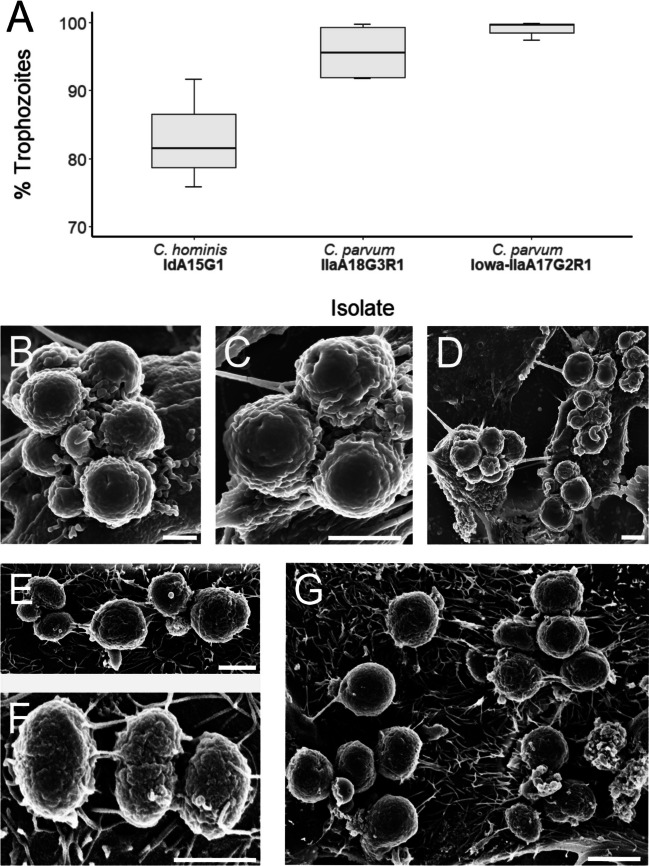
**A** Proportion of trophozoites observed at 48-h post-infection across all three isolates. **B**–**D** Scanning electron micrographs of *C. parvum* (Iowa) trophozoites at 48-h post-infection (scale bars: B =1 μm, C–D = 2 μm). **E**–**G** Scanning electron micrographs of *C. parvum* (clinical isolate) trophozoites at 48-h post-infection (scale bars: 2 μm)

No oocysts or sporozoites were directly observed at either time-point across any of the three subtypes of *Cryptosporidium* that were utilised in this study. Trophozoites were observed in all three *Cryptosporidium* subtypes at both time-points and accounted for the majority of the life cycle stages identified in all cases (Figs. 1A and 2A; Table 2). In *C. hominis* cultures, immature trophozoites were observed to be spherical in shape, with a diameter of approximately 1 μm and a smooth surface (Fig. 1B). Mature trophozoites were observed to be more oblong in shape, 2–4 μm × 2 μm in size and distinctly located extracellularly. Attachment to the host cell through feeder organelles was clearly visible in the mature trophozoites from an extracellular location (Fig. 1C–D). Trophozoites were also observed in end-to-end formation in the *C. hominis* sample at both time-points, although no connecting discs between the two parasites were visible, and observations of this phenomenon were rare (Fig. 1E).

**Table 2:** Median proportion and interquartile range of each observed life cycle stage from each subtype of *Cryptosporidium* (*n* = 3) per time-point

Sample	Time-point	% Trophozoites	% Meronts	% Merozoites	% Macrogamonts	% Microgamonts
*C. hominis* (IdA15G1)	24 h	94.24 (IQR 93.20–95.83)	1.33 (IQR 0.96–1.41)	4.33 (IQR 3.13–5.38)	0.00 (IQR 0.00–0.00)	0.00 (IQR 0.00–0.00)
*C. parvum* (IIaA18G3R1)	24 h	93.95 (IQR 92.10–94.72)	2.53 (IQR 1.60–4.64)	2.35 (IQR 1.68–3.36)	0.14 (IQR 0.00–0.87)	0.00 (IQR 0.00–0.00)
*C. parvum* (Iowa-IIaA17G2R1)	24 h	99.28 (IQR 99.20–99.37)	0.58 (IQR 0.43–0.73)	0.14 (IQR 0.07–0.21)	0.00 (IQR 0.00–0.00)	0.00 (IQR 0.00–0.00)
*C. hominis* (IdA15G1)	48 h	81.50 (IQR 78.66–86.56)	0.00 (IQR 0.00–0.91)	2.72 (IQR 1.67–5.73)	13.15 (IQR 10.46–14.29)	0.00 (IQR 0.00–0.40)
*C. parvum* (IIaA18G3R1)	48 h	95.55 (IQR 91.94–99.26)	1.54 (IQR 0.00–3.32)	0.45 (IQR 0.00–1.30)	1.28 (IQR 0.63–2.01)	0.07 (IQR 0.00–0.42)
*C. parvum* (Iowa-IIaA17G2R1)	48 h	99.56 (IQR 98.49–99.69)	0.00 (IQR 0.00–0.00)	0.35 (IQR 0.17–0.83)	0.19 (IQR 0.14–0.69)	0.00 (IQR 0.00–0.04)

The *C. parvum* (clinical isolate and Iowa) trophozoites were frequently observed in aggregates of two or more at both time-points (Fig. 2B–G). The *C. parvum* (Iowa) trophozoites were spherical in shape, approximately 2 μm in diameter and were relatively uniform in shape and size. In contrast to *C. parvum* (Iowa) trophozoites, the *C. parvum* (clinical isolate) trophozoites were heterogenous in shape and size. They were observed in some instances to be spherical in shape and approximately 2 μm in diameter (Fig. 2E, G), and in other occurrences, they were observed to be oblong in shape and approximately 2–3 μm × 1–2 μm in size (Fig. 2F).

By 48-h post-infection, *C. hominis* trophozoites accounted for a lower proportion of total individuals observed in comparison to the *C. parvum* subtypes (Fig. 2A), *H*(2) = 6.3, *p* = 0.043. *Post hoc* two-tailed multiple comparison tests indicated that this difference was statistically significant when compared to *C. parvum* (Iowa, *difference* = 6.00) where the critical difference (*α* = 0.05 corrected for number of tests) was 5.54. This difference was not statistically significant when compared to *C. parvum* (clinical isolate, *difference* = 4.25) where the critical difference (*α* = 0.05 corrected for number of tests) was 5.18.

**Fig. 3:**
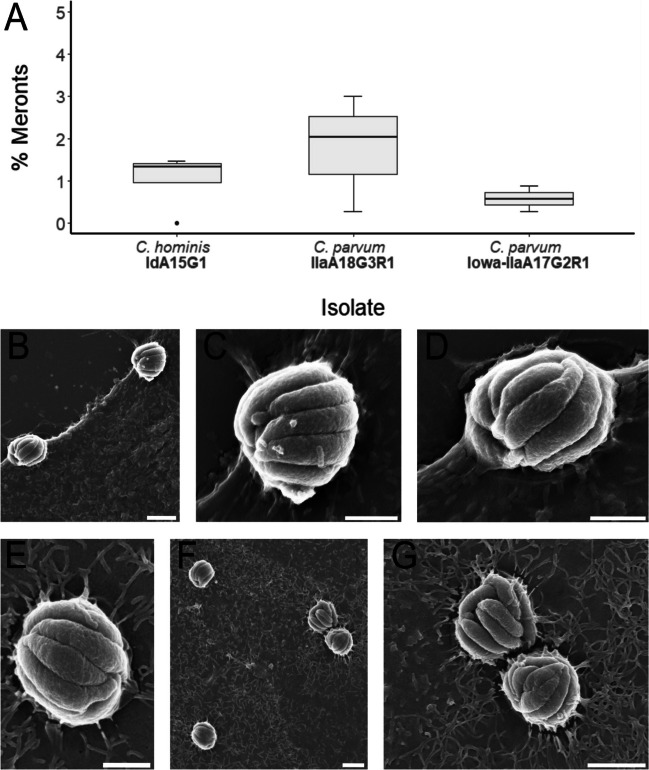
**A** Proportion of meronts observed at 24-h post-infection across all three isolates. **B**–**G** Scanning electron micrographs of *C. hominis* meronts at 24-h post-infection (scale bars: B, F, G = 2 μm; C, D, E = 1 μm)

**Fig. 4:**
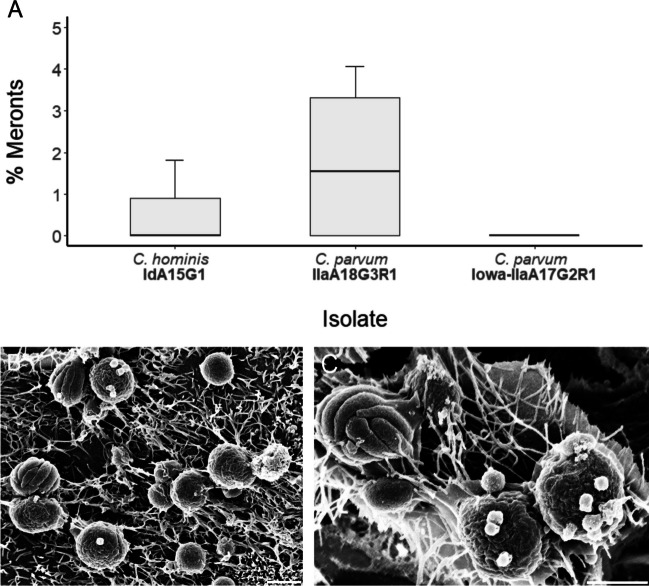
**A** Proportion of meronts observed at 48-h post-infection across all three isolates. **B**–**C** Scanning electron micrographs of *C. parvum* (clinical isolate) meronts at 48-h post-infection (scale bars: 2 μm)

Meronts were infrequently observed in all subtypes at 24-h post-infection (Fig. 3A), and by 48-h post-infection, they were observed in *C. hominis* and *C. parvum* (clinical isolate) only (Fig. 4A). No statistically significant differences in the proportion of meronts between each subtype were observed at 24-h post-infection, *H*(2) = 3.35, *p* = 0.187, or 48-h post-infection, *H*(2) = 2.16, *p* = 0.340. In *C. hominis* cultures, meronts were 2.0–3.0 μm × 2.0–2.5 μm in size and distinct in appearance, with the borders of each internal merozoite clearly visible. The number of internal merozoites visible in each electron micrograph varied between six and eight. The meronts were often observed in close proximity to one another (Fig. 3B, F, G). In the case of *C. parvum* (clinical isolate and Iowa), meronts were approximately 2.0–2.5 × 2.5–3.0 μm in size with between four and seven internal merozoites visible from the host cell surface. These meronts were commonly found in close proximity to each other, as well as to clusters of trophozoites (Fig. 4B–C).

**Fig. 5:**
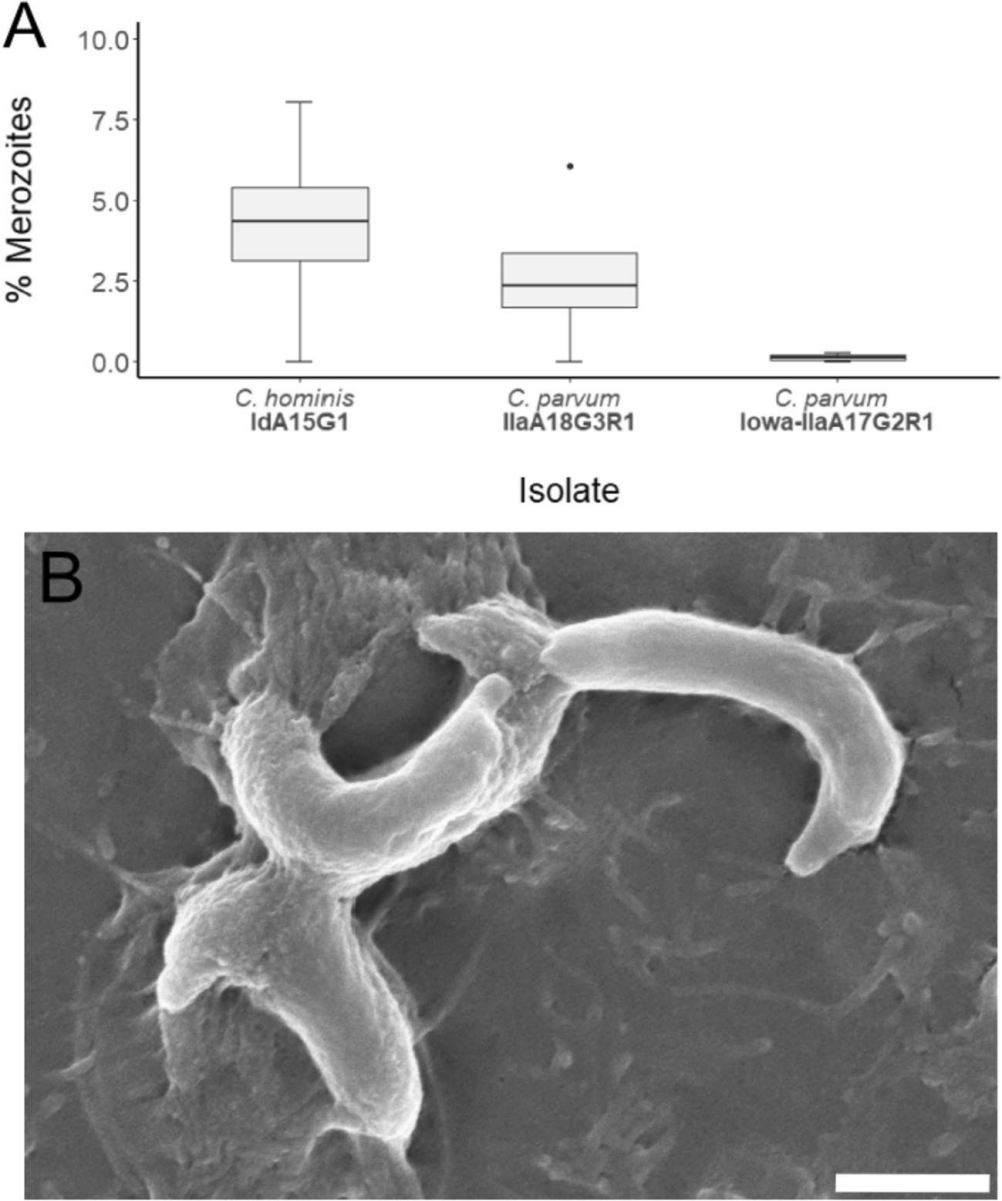
**A** Proportion of merozoites observed at 24-h post-infection across all three isolates. **B** Scanning electron micrograph of *C. hominis* merozoites at 24-h post-infection (scale bar: 1 μm)

**Fig. 6:**
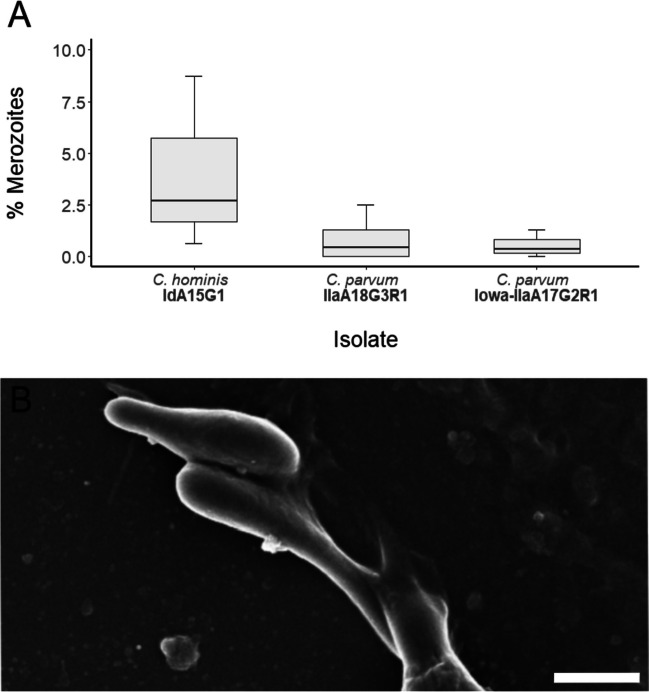
**A** Proportion of merozoites observed at 48-h post-infection across all three isolates. **B** Scanning electron micrograph of *C. hominis* merozoites at 48-h post-infection (scale bar: 2 μm)

Merozoites were identified in *C. hominis*, *C. parvum* (Iowa), and *C. parvum* (clinical isolate) cultures at 24-h post-infection (Fig. 5) and 48-h post-infection (Fig. 6). There was no significant differences in the proportion of merozoites between subtypes at 24-h post-infection, *H*(2) = 3.00, *p* = 0.351, or at 48-h post-infection, *H*(2) = 3.00, *p* = 0.223. The merozoites observed were approximately 3.0 μm in length × 0.5 μm in width, with each merozoite displaying well-defined apical regions (Figs. 5B and 6B).

**Fig. 7:**
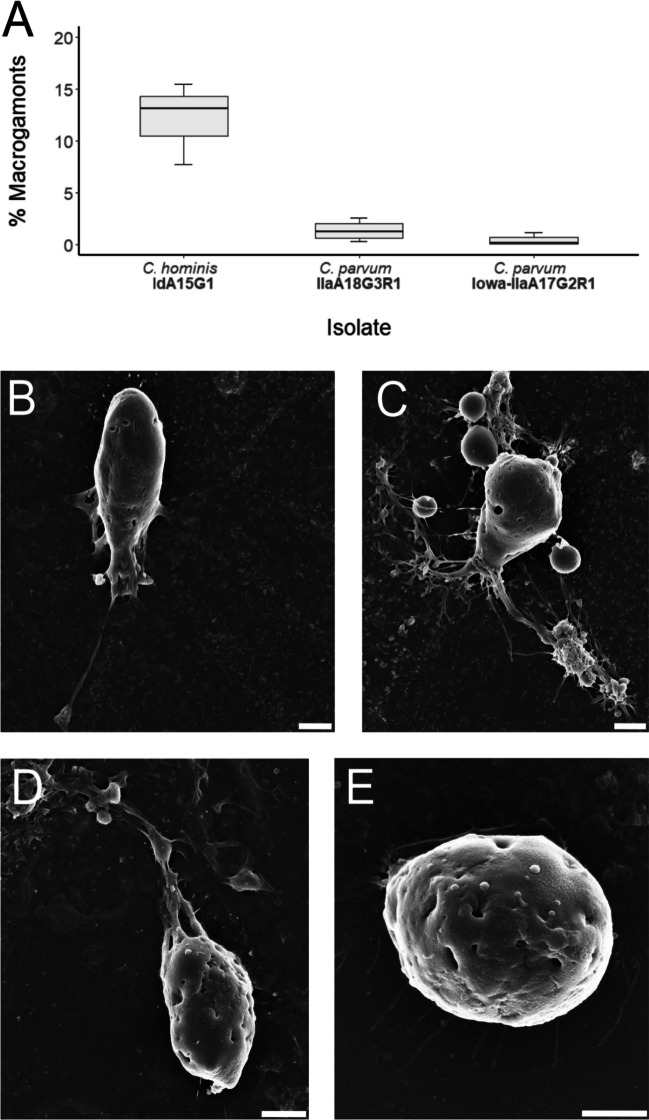
**A** Proportion of macrogamonts observed at 48-h post-infection across all three isolates. **B**–**E** Scanning electron micrographs of *C. hominis* macrogamonts at 48-h post-infection (scale bars: 2 μm)

Macrogamont-like structures were infrequently observed in *C. parvum* (clinical isolate) and *C. parvum* (Iowa) at 48-h post-infection, but observations of this life cycle stage were relatively more frequent in *C. hominis* cultures at the same time-point (Fig. 7). The difference in proportion of macrogamonts between samples was significant, *H*(2) = 6.75, *p* = 0.034. *Post-hoc* two-tailed multiple comparison tests indicated that this difference was only statistically significant when compared to *C. parvum* (Iowa, difference = 6.33) where the critical difference (*α* = 0.05 corrected for number of tests) was 5.54. This difference was not statistically significant when compared to *C. parvum* (clinical isolate, difference = 4.00) where the critical difference (*α* = 0.05 corrected for number of tests) was 5.18.

The morphology of each observed macrogamont was highly heterogenous even within cultures of the same isolate. Trophozoite-like structures were occasionally observed attached to the surface of the macrogamont (Fig. 7C–D). While clear attachment zones to the host monolayer were observed, a distinct feeder organelle was not evident among many of the macrogamonts. The size of each macrogamont varied between 8 and 12 μm × 4 and 6 μm (Fig. 7B–E).

**Fig. 8:**
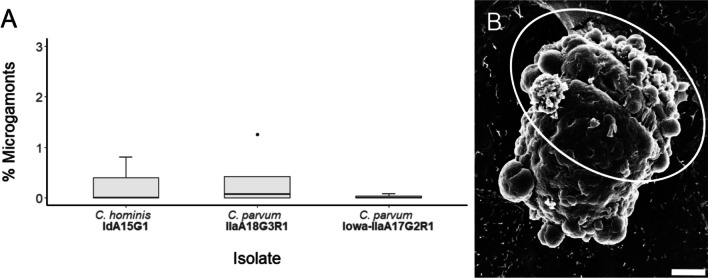
**A** Proportion of microgamonts observed at 48-h post-infection across all three isolates. **B** Scanning electron micrograph of *C. parvum* (clinical isolate) microgamont at 48-h post-infection with microgametes indicated by the circle (scale bar: 2 μm)

Microgamonts were rare but present at 48-h post-infection across the three subtypes of *Cryptosporidium* utilised in this study (Fig. 8A). No significant differences were observed in proportion of microgamonts observed between the different *Cryptosporidium* subtypes, *H*(2) = 0.612, *p* = 0.737. The microgamont shown in Fig. 8B measured approximately 10 μm in length × 8 μm in width, and was observed to be hanging from a stalk with microgametes budding from the surface.

## Discussion

The present study aimed to examine and compare the in vitro growth characteristics of *C. parvum* (Iowa-IIaA17G2R1), *C. parvum* (IIaA18G3R1), and *C. hominis* (IdA15G1) using data acquired via SEM to quantify life cycle stages based on parasite morphology. At 24-h post-infection, predominantly asexual development was observed across all three subtypes of *Cryptosporidium* utilised in this study. The key finding of this study was the observation that *C. hominis* displayed a higher number of sexual life cycle stages at 48-h post-infection when compared to *C. parvum* (Iowa) and *C. parvum* (clinical isolate) at the same time point, and that this was statistically significant when compared to *C. parvum* (Iowa).

*Cryptosporidium hominis* and *C. parvum* are morphologically indistinguishable by light microscopy (Morgan-Ryan et al. 2002), and share a high level of genomic sequence similarity (~97%) (Arias-Agudelo et al. 2020; Guo et al. 2015). Earlier in vitro studies have reported species-specific differences in the in vitro growth characteristics between *C. parvum* and *C. hominis*, such as the ability for *C. parvum* to infect both bovine and human-derived primary intestinal epithelial cells, in contrast to *C. hominis* producing infection in the human-derived primary intestinal epithelial cells only (Hashim et al. 2004). The same study also reported that *C. hominis* (5942 and TU502) infected HCT-8 cell lines less efficiently and less uniformly than *C. parvum* (Iowa) (Hashim et al. 2004). In contrast, other studies have reported minimal differences in the life cycle of *C. parvum* and *C. hominis* in HCT-8 cells, with the exception that *C. hominis* completed its life cycle more rapidly than *C. parvum* (72 h vs. 5 days) (Hijjawi et al. 2001). In the present study, there were no significant differences in the monolayer infection patterns between *C. hominis* and *C. parvum*, but clear differences in life cycle progression were observed.

### Sporozoites

In the present study, sporozoites were not visible in the *C. parvum* or *C. hominis* cultures at either 24-h or 48-h post-infection; an observation which was unsurprising due to the rapid sequence of events in which sporozoite invasion occurs. Sporozoite invasion of the host cell begins within seconds of oocyst excystation (Forney et al. 1999; Guérin et al. 2021; Wetzel et al. 2005). Upon exit from the oocyst, sporozoites exhibit an actin and myosin-dependent gliding motility (Forney et al. 1998; Wetzel et al. 2005), making helical movements prior to productive host cell invasion (Wetzel et al. 2005).

### Trophozoites

Invasive sporozoites transform into replicative trophozoites, and in the in vitro setting, this has been shown to be triggered by proteins in foetal bovine serum, the secretome of HCT-8 cells, and by Gal-GalNAc (Edwinson et al. 2016). The MEDLE-2 secreted protein is exported from the trophozoite into the host cell cytoplasm, where it has been demonstrated to induce an endoplasmic reticulum stress response in the host cell (Dumaine et al. 2021). In the present study, *C. parvum* and *C. hominis* trophozoites were identified at 24-h and 48-h post-infection and represented the most frequently observed life cycle stage at both time-points across all *Cryptosporidium* subtypes. Trophozoites were variable in size ranging from ~1 μm in diameter to larger trophozoites (>2 μm in diameter). Larger *C. parvum* (Iowa) trophozoites were observed to be spherical and relatively uniform in shape and size, whereas *C. parvum* (clinical isolate) and *C. hominis* trophozoites exhibited more heterogeneity in appearance. Smaller trophozoites were often found to have smooth surfaces, which indicate that they were located within the parasitophorous vacuole. Borowski et al. (2010) consistently described *C. parvum* trophozoites to be spherical in shape, regardless of size and relative maturity, which is in line with our observations of *C. parvum* (Iowa). Species-specific differences in *Cryptosporidium* trophozoite maturation have been described previously, where the HCT-8 secretome stimulated *C. parvum* (Iowa) trophozoite development but had minimal effect on *C. hominis* (TU502) development (Edwinson et al. 2016). In the present study, larger *C. hominis* trophozoites were observed to be attached to the host cell via a feeder organelle, which is consistent with morphological and ultrastructural studies on *C. parvum* (Borowski et al. 2010; Huang et al. 2004). Additionally, trophozoites from all *Cryptosporidium* isolates utilised in this study were frequently observed in aggregates of two or more, in line with observations where abundant extracellular accumulations of trophozoites were described (Borowski et al. 2010; Hijjawi et al. 2010; Hijjawi et al. 2004; Rosales et al. 2005). The broader biological significance of why some trophozoites appear located within the parasitophorous vacuole, while others appear extracellular, is currently unknown.

### Meronts and merozoites

In the present study, *C. parvum* and *C. hominis* trophozoites were identified at 24-h and 48-h post-infection and represented the highest proportion of life cycle stages at both time-points across all *Cryptosporidium* subtypes. The proportion of trophozoites observed across the two *C. parvum* subtypes remained the same between the 24-h and 48-h time-points, whereas in the case of *C. hominis*, the proportion of trophozoites observed had dropped considerably by 48-h post-infection. Across both species and all subtypes, each meront was found attached to the apical surface of the host cell membrane, which contrasted with previous observations where *C. parvum* meronts were described to be engulfed by the host cell apical membrane (Borowski et al. 2010). In agreement with previous work (Borowski et al. 2010), internal *C. hominis* merozoites occurred in numbers of six or eight, and meronts were never observed to appear perforated to facilitate merozoite release. There appeared to be more diversity in the number of internal merozoites observed within *C. parvum* (clinical isolate) meronts, with numbers as varied as four and seven. The merozoites inside *C. parvum* meronts have been observed to be aligned solely in parallel orientation (Borowski et al. 2010); however, this was not exclusively the case with the meronts observed across all subtypes of *Cryptosporidium* in the present study, which were infrequently observed to be aligned perpendicularly.

Meronts release motile merozoites that infect new HCT-8 cells and repeat the asexual replication cycle. Studies on *C. parvum* have reported merozoite release to occur between 12 and 18-h post-infection (English et al. 2022; Guérin et al. 2021; Jumani et al. 2019). In the present study, free merozoites were identified across both species and all subtypes at 24-h and 48-h post-infection and were observed to have a tubular shape and well-defined apical regions. Rod-shaped merozoites with pointed apical ends were reported previously at 24-h post-infection in *C. parvum* and were observed to adhere to host cells along their full body length and increase in width throughout invasion (Borowski et al. 2010), which was also the case in the current study.

### Macrogamonts and microgamonts

Notable differences in the abundance of macrogamonts at 48-h post-infection between the two species and three subtypes were evident, where *C. hominis* macrogamonts were frequently identified but were uncommonly observed in either *C. parvum* isolate. Despite the numerical difference between all species as demonstrated in Fig. 7A, this difference was statistically significant when compared to *C. parvum* (Iowa), but not in *C. parvum* (clinical isolate), which was likely due to insufficient number of independent biological replicates to form a statistical model. The size range of the *C. hominis* macrogamonts in the present study was between 8-12 μm × 4–6 μm, which raised the question as to whether some of these larger macrogamont-like structures were actually extracellular gamont-like stages (Hijjawi et al. 2004; Hijjawi et al. 2002). In the case of *C. parvum*, macrogamonts have been described to have less contact with the host cell than the asexual life cycle stages (Borowski et al. 2010), which is in agreement with our findings.

Microgamonts were observed at 48-h post-infection in all three subtypes but they occurred very rarely (summed to nine observations out of 9926 in total across all three subtypes). In *C. parvum*, microgamonts have been described to be smaller than macrogamonts, full of microgametes, and frequently attached to the host cell monolayer via a stalk. Microgamonts had not previously been observed in *C. parvum* cultures until four days post-infection (Borowski et al. 2010). The *C. parvum* (clinical isolate) microgamont shown in our data was morphologically similar to microgamonts already documented in the literature (Koh et al. 2014; Pinto et al. 2022).

Using a genetically engineered *C. parvum* Iowa II strain, a recent study conducted live imaging of the *C. parvum* life cycle in HCT-8 cultures and reported three rounds of asexual meront production, followed by a single generation of gametes (Tandel et al. 2019). In that study, sexual *C. parvum* stages were observed at 36 h and represented approximately 28% of all stages by 48 h and 80% of all stages by 72 h (Tandel et al. 2019). Had sampling continued to 72 h in the present study, it is highly likely more *C. parvum* sexual stages would have been observed. In the study by Tandel et al. (2019), direct development of both macrogamonts and microgamonts was observed from type I meronts containing eight merozoites, and no evidence of type II meronts containing four merozoites was observed, which is consistent with Tyzzer’s original description (Tyzzer 1910). In the present study, we observed variable numbers of merozoites within meronts, but given that we were only able to visualise the apical surface of the infected cell monolayer, the total number of merozoites within the meront may have been higher.

### Oocysts

In the present study, no excysted oocyst shells were observed on the host cell monolayer at either 24-h or 48-h post-infection for *C. parvum* and *C. hominis*. This finding was expected as *Cryptosporidium* oocysts have low attachment efficiency due to the presence of a thick layer of acidic glycoproteins on the outer surface of the oocysts (Kuznar and Elimelech 2006; Liu et al. 2010) and were therefore unlikely to be detected. The binding of *Cryptosporidium* oocysts to surfaces is highly variable between different species and influenced by oocyst age (Sarkhosh et al. 2019) but excysted *C. parvum* oocyst shells have been reported to adhere to the host cell surface at 24-h post-infection in a similar in vitro culturing system (Borowski et al. 2010), suggesting that the detection of oocyst shells on the host cell surface was unlikely but still possible. Tandel et al. (2019) reported that fertilisation of *C. parvum* gametes in HCT-8 cell culture is limited or absent with no new oocysts produced. Future studies on the *C. hominis* life cycle in vitro will confirm if fertilisation is also absent or minimal in this species in static culture.

## Conclusions

This study provided a comparative analysis of the in vitro growth characteristics of *C. hominis* (IdA15G1), *C. parvum* (IIaA18G3R1), and *C. parvum* (Iowa-IIaA17G2R1). Our systematic SEM observations showed that *C. hominis* yielded a notably higher proportion of macrogamonts at 48-h post-infection, indicating higher levels of sexual development at this time-point when compared to both subtypes of *C. parvum* that were utilised in this study. This difference was statistically significant when compared to *C. parvum* (Iowa) but was not significant in the case of *C. parvum* (clinical isolate). This was also reflected in statistically significant differences in proportion of trophozoites between *C. hominis* and *C. parvum* (Iowa) at 48-h post-infection. No significant differences in the proportion of meronts or merozoites were measured at either time-point, nor was the frequency of microgamont observations at 48-h post-infection significantly different between any of the three subtypes of *Cryptosporidium* that were utilised in this study.

A limitation of the present study is that the findings and conclusions are based only on the HCT-8 transwell system, given that the in vitro development of *Cryptosporidium* may differ under alternative culture conditions using different cell types. Additionally, samples were only analysed at 24 h and 48 h. A more complete comparison of the life cycle characteristics of *C. parvum* and *C. hominis* would have been achieved by analysis at a greater number of time-points and continuing the evaluation until at least 72 h. Furthermore, while oocysts no older than 12 weeks were used in the experiments described herein, the data collected in this study did not incorporate the relative oocyst age of each isolate into the analyses and the impact this may have had on *Cryptosporidium* development. The findings of this study justify further investigation of differences of *in* vitro growth characteristics between *C. parvum* and *C. hominis* and should focus on an increased number of time-points over an extended period. Further avenues for future studies may include assessment of the growth characteristics of a larger number of *C. parvum* and *C. hominis* subtypes with a greater number of independent biological replicates in HCT-8 cultures and examination of the supernatant for oocysts. This study has advanced our understanding of the in vitro development of *C. hominis* and provided insights into how it may contrast with *C. parvum*.

## Data Availability

Further information and requests for materials should be directed to the corresponding author.
